# The effects of a 12-week worksite physical activity intervention on anthropometric indices, blood pressure indices, and plasma biomarkers of cardiovascular disease risk among university employees

**DOI:** 10.1186/s13104-018-3151-x

**Published:** 2018-01-29

**Authors:** Duane B. Corbett, Curtis Fennell, Kylene Peroutky, J. Derek Kingsley, Ellen L. Glickman

**Affiliations:** 1 Department of Aging and Geriatric Research, College of Medicine, University of Florida, Gainesville, FL 32611 USA; 2 Department of Kinesiology, College of Education, University of Montevallo, Montevallo, AL 35115 USA; 3 Department of Exercise Physiology, School of Health Sciences, Kent State University, Kent, OH 44240 USA

**Keywords:** Sedentary behavior, Exercise, Physical inactivity, Workplace, Health promotion

## Abstract

**Background:**

To determine the effectiveness of a low-cost 12-week worksite physical activity intervention targeting a goal of 10,000 steps per day on reducing anthropometric indices, blood pressure indices, and plasma biomarkers of cardiovascular disease (CVD) risk among the employees of a major university.

**Methods:**

Fifty university employees (*n* = 43 female, *n* = 7 male; mean age = 48 ± 10 years) participated in the 12-week physical activity intervention (60 min, 3 day/week). Each session included both aerobic (cardiorespiratory endurance) and muscle-strengthening (resistance) physical activity using existing university facilities and equipment. Anthropometric indices, blood pressure indices, and plasma biomarkers of CVD risk assessed included those for obesity (body mass index), hypertension (systolic blood pressure, SBP; diastolic blood pressure, DBP), dyslipidemia (high-density lipoprotein, HDL; low-density lipoprotein, LDL; total serum cholesterol), and prediabetes (impaired fasting glucose, IFG). Steps per day were assessed using a wrist-worn activity monitor. Participants were given the goal of 10,000 steps per day and categorized as either compliers (≥ 10,000 steps per day on average) or non-compliers (< 10,000 steps per day on average) based on their ability to achieve this goal.

**Results:**

Overall, 34% of participants at baseline were already at an elevated risk of CVD due to age. On average, 28% of participants adhered to the goal of 10,000 steps per day. After 12-weeks, participants in both groups (compliers and non-compliers) had lower BMI scores (p < 0.001), lower HDL scores (p < 0.034), and higher IFG scores (p < 0.001). The non-compliers had a greater reduction of BMI scores than the compliers (p = 0.003). Participants at risk for CVD had greater reductions than those not at risk for several risk factors, including SBP (p = 0.020), DBP (p = 0.028), IFG (p = 0.002), LDL (p = 0.006), and total serum cholesterol (p = 0.009).

**Conclusion:**

While the physical activity intervention showed mixed results overall with both favorable changes in anthropometric indices yet unfavorable changes in plasma biomarkers, it was particularly beneficial in regards to both blood pressure indices and plasma biomarkers among those already at risk of CVD.

*Trial registration* ClinicalTrials.gov NCT03385447; retrospectively registered

## Background

Physical inactivity is well established as a leading preventable cause of cardiovascular disease (CVD)—the leading cause of death and a major contributor to rising healthcare costs in the United States [1, 2]. In fact, people who are physically inactive spend 38% more days hospitalized compared to those who are physically active [3]. While the paramount importance of increasing physical activity is to improve quality of life and longevity, it is also a critical factor in the effort to reduce the continually increasing national healthcare burden. With the declining prevalence of adults with no known major CVD risk factors, the national healthcare burden of CVD is forecasted to triple by the year 2030 [4]. This information is a major concern to employers, who on average pay 72% of continually increasing annual health coverage premiums [5]. Institutions of higher education, whose workforce is predominantly limited to sedentary labor, may have more concern for this information [6]. Taking advantage of the recent surge in wearable technology that makes personal fitness tracking an affordable reality [7], this study examines the possibility of reducing the risk of CVD through a low-cost, goal-based worksite physical activity intervention in a university setting.

Worksite physical activity interventions represent an attractive, cost-effective investment for employers through improved healthcare costs, rates of absenteeism, and worker productivity [8, 9]. While eliminating many of the barriers that prevent adults from being physically active in the first place (e.g., lack of social support, limited access to resources) [10–12], a worksite physical activity intervention in the university setting—a traditionally underrepresented population [13]—also provides the unique environmental opportunity to minimize start-up costs through use of existing facilities and equipment (e.g., gymnasiums, health and physical education equipment) [14]. Furthermore, the ability to hold such an intervention in a location separate from younger adults (i.e., students), such as an annex gymnasium that is not part of student recreation, may improve group cohesiveness and physical activity participation compared to other physical activity options with intermixed age groups [15].

As mentioned previously, the recent advancement of wearable technology provides affordable options for the objective monitoring of physical activity [7]. While the benefit of activity monitors has traditionally been limited to researchers due to their prohibitively high cost, advances in technology have now drastically increased their consumer accessibility. As such, it is now possible for virtually anyone to self-monitor their physical activity through wearables (e.g., pedometers). Since there is limited literature on the benefit of wearables, a worksite physical activity intervention designed to reduce CVD risk that includes wearables is intriguing. In fact, a meta-analysis of 32 studies showed the use of wearables had a moderate and positive effect on increasing physical activity, although the direct measure of CVD risk was not included in the analysis [16]. Interestingly, studies in this analysis that included a goal of 10,000 steps per day had the greatest effect. Therefore, including a goal of 10,000 steps per day may be an important component when designing a worksite physical activity intervention aimed at reducing CVD risk.

In view of these considerations, the purpose of the following study was to examine the effects of a 12-week low-cost, goal-based worksite physical activity intervention on anthropometric indices, blood pressure indices, and plasma biomarkers for risk of CVD—the leading cause of death and disability in the United States [17]—among faculty and staff members at a major university. In addition, we also examined the effects of providing participants with a low-cost physical activity monitor to self-monitor their effort throughout the duration of the intervention. As such, we hypothesized that a low-cost worksite physical activity intervention consisting of both aerobic and anaerobic physical activity that targets the goal of 10,000 steps per day would improve indices and biomarkers of CVD risk among university employees. We also hypothesized that intervention participants who adhered to the goal of 10,000 steps per day would have greater improvements in indices and biomarkers of CVD risk than those who did not adhere. Finally, we hypothesized that individuals at risk for CVD through specific indices and biomarkers, would have greater improvements in those indices and biomarkers than those not at risk.

## Methods

### Participants

Fifty university employees (*n* = 43 females; 28–65 years) were recruited for the physical activity intervention and participation in the study. Enrollment was based on a convenience sample with recruitment achieved through university mass email in addition to university newspaper and website advertising. Primary inclusion criteria limited participants to university faculty and staff members who were self-reported to be sedentary with no contraindications to physical activity prior to participation in the intervention as reported via a questionnaire (Physical Activity Readiness Questionnaire) and physician release form, respectively. The study was approved by the Kent State University Institutional Review Board and all participants gave their informed consent in writing.

### Physical activity intervention

The physical activity intervention targeted the current federal physical activity guidelines recommendations for adults [18], consisting of 60-min sessions of both aerobic (cardiorespiratory endurance) and anaerobic (muscle strengthening resistance) physical activity, 3 days/week with a day of rest between each session, for a period of 12-weeks. The intervention was offered as both a morning (6 a.m.) and noon (12 p.m.) session with participation restricted to one session per day. For each session, participants reported to the exercise physiology laboratory which included multiple gymnasiums and equipment for group-based activities. These gymnasiums were independent of the student recreation center, which allows employee participants to engage in physical activity at a separate environment than the students. The 60-min sessions included 5-min warm-up and cool-down periods. A variety of instructor-led group-based physical activity choices were offered during each session. These choices included, but were not limited to, group walking and running, aerobic dancing, yoga, basketball, dodgeball, badminton, and various boot-camp style classes. In addition, participants were offered a more independent alternative which included a room with cardiovascular equipment and fixed-weight machines. Previous work shows that having a greater variety of activities may enhance adherence through increased enjoyment and decreased boredom [19, 20]. Participants were encouraged to progress to more challenging activities as the intervention continued. All activities were instructed, supervised, and monitored by trained exercise specialists. All facilities used by the intervention were during periods of non-conflict with university courses. All equipment used for the intervention were pre-owned by departments within the Kent State University College of Education, Health, and Human Services and were not being used otherwise.

### Measurements

Assessments were conducted during laboratory visits at baseline and at the end of 12-weeks. All assessments were conducted by certified exercise physiologists or trained phlebotomists. Assessments included self-reported demographic and contact information, anthropometric indices for CVD risk, blood pressure indices for CVD risk, and plasma biomarkers for CVD risk. Anthropometric indices, blood pressure indices, and plasma biomarkers assessed were in accordance to current criteria outlined by the American College of Sports Medicine [21]. Anthropometric indices assessed included body mass index (BMI)—a marker for obesity—calculated as weight in kilograms divided by height in meters squared and scored as ≥ 30 kg/m^2^ for both men and women. Blood pressure indices were non-gender specific and included systolic blood pressure (SBP) and diastolic blood pressure (DBP)—markers for hypertension—scored as ≥ 140 or ≥ 90 mmHg, respectively. Plasma biomarkers assessed were also non-gender specific and included high-density lipoprotein (HDL), low-density lipoprotein (LDL), and total serum cholesterol—markers for dyslipidemia—scored as < 40, ≥ 130, and ≥ 200 mg/dL, respectively; and impaired fasting glucose (IFG)—a marker for prediabetes—scored as ≥ 100 and ≤ 125 mg/dL. Participants were required to fast to at least 8 h prior to having blood drawn.

In addition to the assessments done during laboratory visits to establish risk factors for CVD, each participant was given a Movband activity monitor (Movable, Cleveland, OH). The Movband is a low-cost commercially available wrist-worn activity monitor that features a tri-axial accelerometer to assess daily step count. The device also features a liquid crystal display screen that allows the user to monitor their step count in real-time. In-house preliminary data suggest the Movband to be a valid measure of free-living physical activity [22]. Participants were instructed to wear the activity monitors on their non-dominant wrist each day during periods of wake throughout the duration of the study with a goal of 10,000 steps per day. Participants were categorized as either compliers (≥ 10,000 steps per day) or non-compliers (< 10,000 steps per day) based on their ability to achieve the 10,000 steps per day goal on average over the duration of the study. The 10,000 steps per day cut-point was chosen based on previously proposed indices that suggest this level to be a reasonable estimate of recommended daily physical activity for apparently healthy adults [23]. Non-wear days were defined as having fewer than 200 steps recorded and were not included in the analysis. Individuals with seven or more non-wear days were excluded from the steps per day compliance analysis. Attendance was recorded daily through login sheets upon arrival to the facility.

### Data analysis

All data analyses were conducted using Stata 13.1 (Stata Corp, College Station, TX). Baseline participant characteristics were compared across compliance groups using independent samples t-tests and Chi square tests for categorical variables. Measures for CVD risk at both baseline and follow-up (12-weeks) were compared overall using paired samples t-tests and across compliance baseline risk groups using independent samples t-tests with pre-post values. Participant characteristics and measures for CVD risk at both baseline and follow-up (12-weeks) are summarized in table form (mean, SD; *n*, %). Average daily step count was assessed for each participant as an average of the weekly average for all 12 weeks of the study. Attendance was assessed as a total percentage for each participant, averaged as both the entire sample and by compliance group. For this study, enrollment was limited to a small convenience sample and therefore a sample size calculation was not necessary. Alpha was set a priori at p ≤ 0.05.

## Results

### Baseline characteristics

Participant characteristics at baseline, attendance, and average steps per day are presented in Table 1. Baseline characteristics were similar between the two groups; however, the compliers had significantly higher HDL scores (p = 0. 029) and average steps per day (p < 0.001) compared to the non-compliers. Overall, 34% of participants at baseline were already at an elevated risk of CVD due to age. Detailed statistics for anthropometric indices, blood pressure indices, and plasma biomarkers of CVD risk are presented in Tables 2 and 3. Based on the baseline data, 30% of participants were already at elevated risk of CVD due to BMI, 16% due to SBP, 6% due to DBP, 8% due to IFG, 36% due to LDL, 16% due to HDL, and 38% due to total serum cholesterol. Accordingly, 24% of all participants were at multiple risk, defined as being at risk of two or more risk factors associated with indices and biomarkers listed (e.g., SBP and DBP only represents one risk) including negative risk for HDL (scored as ≥ 130 mg/dL), but not at risk due to age.

**Table 1:** Characteristics of participants

	Total (*n* = 50)	Participants with activity monitor data	
		Compliers (*n* = 14)	Non-compliers (*n* = 10)
Age (year)	48 (10)	48 (10)	44 (12)
Female, % (*n*)	86 (43)	79 (11)	80.0 (8)
Weight (kg)	93.8 (24.9)	88.6 (15.5)	109.1 (39.2)
BMI (kg/m^2^)	32.7 (7.1)	31.5 (5.9)	37.9 (10.9)
Ethnicity/race, % (*n*)			
White	84 (42)	93 (13)	70 (7)
African American	14 (7)	7 (1)	20 (2)
Other	2 (1)	0.0 (0)	10 (1)
SBP (mmHg)	124.9 (12.6)	124.4 (16.6)	120.8 (8.1)
DBP (mmHg)	75.2 (9.9)	74.9 (9.6)	73 (8.0)
IFG (mg/dL)	89.4 (23.7)	93.4 (16.9)	87.4 (29.6)
LDL (mg/dL)	115.0 (34.4)	107.4 (33.7)	102.2 (33.1)
HDL (mg/dL)	51.2 (12.3)	50.3 (11.1)*	40.8 (7.6)
Total serum cholesterol (mg/dL)	190.8 (39.3)	179.7 (39.9)	173.3 (40.9)
CVD risk due to age, % (*n*)	34.0 (17)	42.9 (6)	30 (3)
Steps per day	10,633.9 (2797.7)	12,418.2 (1863.5)*	8136.0 (1760.8)
Attendance rating, mean % (SD)	65 (20)	75 (18)	63 (20)

Data are means and SD unless otherwise noted. All measures are representative of baseline with the exception of steps per day and attendance percentage being the average over the duration of the study. CVD risk due to age, men ≥ 45 year, women ≥ 55 year; attendance rating, average percentage of classes participated in. *BMI* body mass index, *SBP* systolic blood pressure, *DBP* diastolic blood pressure, *IFG* impaired fasting glucose, *LDL* low-density lipoprotein, *HDL* high-density lipoprotein

* Significant difference from non-compliers at p < 0.05

**Table 2:** Change in anthropometric indices, blood pressure indices, and plasma biomarkers of CVD risk overall and by compliance

Risk factor	Total (*n* = 50)	p value	Participants with activity monitor data		p value
			Compliers (*n* = 14)	Non-compliers (*n* = 10)	
Obesity					
BMI (kg/m^2^)	− 0.59^a^	< 0.001*	− 0.43	− 1.43	0.003*
Hypertension (mmHg)					
SBP	− 0.06^b^	0.977	− 2.31^d^	1.50^e^	0.549
DBP	0.23^b^	0.875	− 1.08^d^	3.70^e^	0.249
Prediabetes					
IFG (mg/dL)	9.84^c^	< 0.001*	9.10	13.50^f^	0.587
Dyslipidemia (mg/dL)					
LDL	0.53^c^	0.887	3.70^e^	0.75^f^	0.650
HDL	− 2.58^c^	0.034*	− 1.20^e^	1.13^f^	0.331
Total serum cholesterol	0.68^c^	0.870	− 2.80^e^	1.75^f^	0.896

Data are presented as delta of means from baseline to 12-weeks

*BMI* body mass index, *SBP* systolic blood pressure, *DBP* diastolic blood pressure, *IFG* impaired fasting glucose, *LDL* low-density lipoprotein, *HDL* high-density lipoprotein

* Significance at p < 0.05

^a^*n* = 49, ^b^ *n* = 48, ^c^ *n* = 38, ^d^ *n* = 13, ^e^ *n* = 10, ^f^ *n* = 8

**Table 3:** Change in anthropometric indices, blood pressure indices, and plasma biomarkers of CVD risk by baseline risk

Risk factor	*n*	Risk	*n*	No-risk	p value
Obesity					
BMI (kg/m^2^)	29	− 0.73	20	− 0.38	0.112
Hypertension (mmHg)					
SBP	7	− 12.29	41	2.02	0.020*
DBP	3	− 12.00	45	1.04	0.028*
Prediabetes					
IFG (mg/dL)	2	− 18.00	36	11.39	0.002*
Dyslipidemia (mg/dL)					
LDL	11	− 15.00	27	6.85	0.006*
HDL	5	0.60	33	− 3.06	0.296
Total serum cholesterol	14	− 13.07	24	8.71	0.009*

Data are presented as delta of means from baseline and 12-weeks follow-up

*BMI* body mass index, *SBP* systolic blood pressure, *DBP* diastolic blood pressure, *IFG* impaired fasting glucose, *LDL* low-density lipoprotein, *HDL* high-density lipoprotein

* Significance at p < 0.05

### Steps per day

**Fig. 1:**
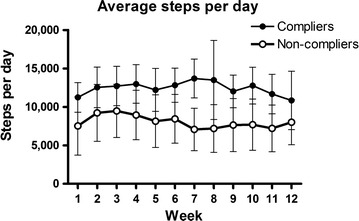
Average steps per day by week by compliance group

Average steps per day by group by week are presented in Fig. 1. Across the duration of the intervention, only 24 participants (48%; n = 14 compliers, n = 10 non-compliers) fulfilled the inclusion criteria for analysis. Average steps per day were significantly higher among the compliers compared to the non-compliers across all weeks of the physical activity intervention (p < 0.05).

### Anthropometric indices, blood pressure indices, and plasma biomarkers of CVD risk

After 12-weeks, all participants had lower BMI scores (pre-post = − 0.59 kg/m^2^, p < 0.001), lower HDL scores (pre-post = − 2.58 mg/dL, p < 0.034), and higher IFG scores (pre-post = 9.48 mg/dL, p < 0.001) after 12-weeks. The non-compliers had a greater reduction in BMI scores than the compliers (non-compliers: pre-post = − 1.43 kg/m^2^, compliers: pre-post = − 0.43 kg/m^2^, p = 0.003). Participants at risk had greater reductions than those not at risk for several risk factors, including SBP (risk: pre-post = − 12.29 mmHg, no-risk: pre-post = 2.02 mmHg, p = 0.020), DBP (risk: pre-post = − 12.00 mmHg, no-risk: pre-post = 1.04 mmHg, p = 0.028), IFG (risk: pre-post = − 18.00 mg/dL, no-risk: pre-post = 11.39 mg/dL, p = 0.002), LDL (risk: pre-post = − 15.00 mg/dL, no-risk: pre-post = 6.85 mg/dL, p = 0.006), and total serum cholesterol (risk: pre-post = − 13.07 mg/dL, no-risk: pre-post = 8.71 mg/dL, p = 0.009). It should be noted that only three participants IFG levels were at or above 125 mg/dL at baseline (pre-post = 1.33 mg/dL).

## Discussion

This study examined the effects of a low-cost, goal-based 12-week worksite physical activity intervention on anthropometric indices, blood pressure indices, and plasma biomarkers of CVD risk among faculty and staff members at a major university. Variables were examined across time overall and by groups separated in two different ways: (1) based on compliance to the goal of 10,000 steps per day using a wrist-worn activity monitor, and (2) baseline risk for CVD for each individual index and biomarker. The use of the activity monitor allowed for objective measurement of daily physical activity for the duration of the study and also provided objective, real-time feedback on personal physical activity levels to the participants.

As expected, overall improvements were found for anthropometric indices with a decrease in BMI scores. Unexpectedly, however, overall plasma biomarkers of CVD risk showed unfavorable reductions in both IFG and HDL levels. The intervention showed no effect on blood pressure indices. Surprisingly, there was no benefit to compliance to the 10,000 steps per day goal due to the non-compliers actually reporting greater improvements in BMI scores than the compliers. For individuals at risk for CVD through baseline assessment according to each specific index and biomarker, the intervention was considerably beneficial for blood pressure indices and plasma biomarkers in comparison to those with no baseline risk. Specifically, participants with baseline risk found greater improvements in SBP, DBP, IFG, LDL, and total cholesterol levels compared to those with no baseline risk for those specific measures.

Although our results may not be of clinical significance, we do report that no participant increased their risk of CVD due to BMI. In addition, we also report that at 12-weeks follow-up, while one participant actually dropped a level within the subcategories of obesity for BMI, three other participants were within the overall average BMI reduction away from dropping BMI as a risk factor for CVD altogether. Regardless, the majority of changes were desirable with 80% of participants having lower BMI scores at 12-weeks follow-up compared to baseline. Similar concern could be raised in regards to our reported change in IFG and HDL levels. However, both IFG and HDL levels are historically prone to having a wide degree of variability, especially in response to exercise training for which an analysis may require control for a variety of potential confounders for which our current analysis could not account [24, 25]. However, our sample size severely limits the statistical power required for such an analysis so we will continue to report only the absolute pre-post values. That said, we would not necessarily interpret the unfavorable reductions in HDL and IFG levels as an increased risk of dyslipidemia or prediabetes.

The findings of this study may have important public health significance. While the majority of worksite physical activity studies have focused on the industrial job sector, previous works shows a relatively equal level of health risk among white-collar workers [26, 27]. In our results, we found promising effects of such an intervention focused in the predominantly white-collar setting of a major university, with desired changes seen in anthropometric indices, blood pressure indices, and plasma biomarkers for CVD risk at 3-months of follow-up. For perspective purposes, we compared our results with a comprehensive meta-analysis of 138 workplace physical activity interventions ranging in duration from 4 to 2028 supervised physical activity sessions, with a similar median duration of 36 sessions (Q1, 28 sessions; Q3, 60 sessions) [26]. In this meta-analysis, diabetes risk was significantly reduced (average IFG pre-post = − 12.6 mg/dL), and changes in lipids and anthropometrics were modest, yet desirable (average total cholesterol/HDL ratio pre-post = − 0.2; average BMI pre-post = − 0.3) [26]. Blood pressure indices were not an included measures of the analysis, however, several studies included in the analysis did account for blood pressure indices with significantly positive results observed [28]. In contrast of these results, our study showed that 3 months of participation in a worksite physical activity intervention is ample duration to elicit similar effects (with the exception of overall HDL and IFG levels). In fact, our results showed greater improvements than those reported in the meta-analysis for BMI scores overall and IFG levels among those already at risk. Important to note, only one study included in the meta-analysis was conducted in a university setting with the results never reaching publication, leading further credence to the importance of the current study.

Our finding that compliance to the goal of 10,000 steps per day had no benefit on CVD risk factors was surprising. While we hypothesized that the addition of a wearable device to self-monitor compliance to the goal of 10,000 steps per day would be beneficial improving CVD risk, our results showed no benefit to compliance with non-compliers even having a greater reduction of CVD risk due to BMI. These findings contradict those of a previous meta-analysis that showed the use of wearables to have positive benefit on increasing physical activity and a goal of 10,000 steps per day to have the greatest benefit [16]. Without having the benefit of knowing the true change in daily physical activity from baseline prior to beginning the intervention, the interpretation of these results should not be limited to face value. In fact, upon closer inspection, an argument could be made that the non-compliers group may have been more physically inactive than the compliers group at the start of the intervention, with evidence of their higher baseline BMI scores and lower HDL levels. As such, perhaps the non-compliers change in daily steps was smaller in comparison to the compliers group in absolute value, yet larger in relative value. Again, we are limited to speculation since steps per day were only monitored once individuals began the intervention. In contrast, it may be so that wearables provide no added benefit to reducing CVD risk. In a randomized clinical trial, it was shown that wearables provided no enhanced benefit to a standard behavioral intervention for weight loss, with participants assigned wearables reporting less weight loss over a 24 month period than those not assigned a wearable [29]. It should be noted, however, that this trial was limited to non-supervised independent physical activity. Still, other work suggests that the successful use of wearables to facilitate health behavior changes may be dependent upon more complex engagement strategies that combine elements of individual encouragement, social competition and collaboration, and effective feedback loops [30].

Strengths of the current study include its relatively heterogeneous sample with respect to age, gender, and ethnicity/race, and the baseline similarity between compliance groups among these variables. In addition, the occupational homogeneity of the sample representative of the non-manual labor work force, the inclusion of the activity monitor allowed for objective measure of daily physical activity over the duration of the study, and the university setting allowed for novelty in our approach to examine a highly underrepresented cohort in worksite physical activity research. The results of the current study should also be interpreted in the context of several limitations. One of the obvious limitations of our study was that it was essentially a pilot study with a small sample size of 50 faculty and staff members, and as such, we did not conduct a power analysis. It was also a limitation that our analysis did not account for the varying intensity of the available activities performed. However, since the focus our analysis was on compliance to the goal of 10,000 steps per day and not adherence to intensity based guidelines, we feel the valid objective measure of physical activity used for this study was reasonable. In addition, the study design did not allow for objective assessment of physical activity prior to starting the intervention, and thus we were unable to examine physical activity at baseline. A similar limitation of our study design was the lack of an appropriate control or comparison group. However, we feel that our categorization of compliers and non-compliers satisfied this consideration to some degree. Lastly, while we make the argument that an attractive feature of a worksite physical activity intervention would be reducing employee healthcare costs, we did not include any healthcare data in our analysis. However, proving an intervention such as the one here can exist in a university setting with minimal start-up and expenses, does make it more attractive through the minimal cost-associated risk involved.

## Conclusions

In summary, the physical activity intervention showed somewhat mixed results for participants overall, with favorable changes in anthropometric indices yet unfavorable changes in plasma indices and essentially no benefit for compliance to the goal of 10,000 steps per day. However, participation was particularly beneficial for those who were already at risk of CVD through baseline assessment according to each specific index and biomarker. In fact, those at risk showed favorable changes both blood pressure indices and plasma biomarkers relative to those not at risk, while also reporting a favorable, although not significant, trend in anthropometric indices. These results suggest that a low-cost worksite physical activity intervention in a university setting is feasible and may be an effective method to reduce employee risk of CVD. As such, these results have implication that such an intervention may be an effective method for reducing employee CVD risk in a university setting without the cost associated-risk of start-up. Further longitudinal study is needed, including employee healthcare expenditure data, to examine the long-term effects of such an intervention.

## Abbreviations

CVD: cardiovascular disease
BMI: body mass index
SBP: systolic blood pressure
DBP: diastolic blood pressure
IFG: impaired fasting glucose
LDL: low-density lipoprotein
HDL: high-density lipoprotein
